# Suicide in DSM-5: Current Evidence for the Proposed Suicide Behavior Disorder and Other Possible Improvements

**DOI:** 10.3389/fpsyt.2020.499980

**Published:** 2021-02-04

**Authors:** Kara B. Fehling, Edward A. Selby

**Affiliations:** ^1^NYCBT, New York, NY, United States; ^2^Department of Psychology, Rutgers, The State University of New Jersey, New Brunswick, NJ, United States

**Keywords:** suicide, suicide attempt, suicidal ideation, suicide behavior disorder, risk

## Abstract

Suicide continues to be one of the greatest challenges faced by mental health clinicians and researchers, an issue made worse by increasing trends in the global suicide rate. Suicide behavior disorder (SBD) was introduced in *DSM-5* as a disorder for further consideration and potential acceptance into the diagnostic system. There are numerous positive developments that would arise from the addition of a suicide-related diagnosis. Utilizing the 2009 guidelines established by Kendler and colleagues, the present review examines the evidence for SBD's validity and discusses the diagnosis' potential clinical benefits and limitations. Altogether, growing evidence indicates that SBD has preliminary validity and benefit. SBD presents with several significant limitations, however, and possible alternative additions to future *DSMs* are highlighted.

## Suicide in DSM-5: Current Evidence for the Proposed Suicide Behavior Disorder and Other Possible Improvements

Suicide is one of the most pressing public health concerns facing modern society, with more than 40,000 people dying by suicide each year in the United States (1), and emerging chronological trends suggest that suicide rates are increasing both within the United States (2) and globally (3). Prevention efforts have proven difficult to develop, possibly because no one risk factor predicts suicide with high accuracy (4). Even suicidal ideation and mental illness, the most commonly cited risk factors, do not always or exclusively predict suicidal behavior (5). Recently, various articles have been written to emphasize the importance of suicide risk assessment in improving suicide prevention (6, 7). One possible way to improve suicide assessment is to include suicidal behavior more thoroughly in universal classification systems of mental disorders.

Accordingly, in recognition of suicide's importance as a psychiatric complication, the fifth edition of the *Diagnostic and statistical manual of mental disorders* [*DSM-5*; (8)] took a major step in suggesting Suicidal Behavior Disorder (SBD) as a “condition for further study.” This proposal means that SBD might be included in a later edition, pending further research. In the *DSM-5* and earlier versions of the manual, suicide is conceptualized primarily as a specific symptom of Major Depressive Disorder (MDD) and Borderline Personality Disorder (BPD), or as a possible negative consequence of other psychiatric diagnoses (8). In addition to research, critical discussion is needed to determine whether SBD is a valid and clinically useful diagnosis to embrace. Fortunately, the APA has devised specific recommendations that guide *DSM* diagnostic changes, additions, and removals (9). Here, we review these guidelines and evaluate the extent to which SBD meets these guidelines based on existing research on suicide. Furthermore, we argue that in its present form, *DSM-5* does a disservice to the field in the way it includes (and doesn't include) suicide, and we discuss ways in which the next *DSM* could be improved regardless of SBD's presence.

## Proposal of a New Suicide Diagnosis

SBD is one of eight conditions for further study that was included in Section III of the *DSM-5*. Along with the other proposed disorders, SBD criteria were determined by seasoned experts on the *DSM-5* Task Force and Work Groups by comprehensively examining the research literature and discussing the criteria with the field and general public (8). As proposed currently, a diagnosis of SBD would require an individual to meet all five of five of the following diagnostic criteria:

A. Within the last 24 months, the individual has made a suicide attempt.
B. The act does not meet criteria for non-suicidal self-injury (NSSI).
C. The diagnosis is not applied to suicidal ideation or to preparatory acts.
D. The act was not initiated during a state of delirium or confusion.
E. The act was not undertaken solely for a political or religious objective.

The proposed diagnosis includes two specifiers: “current” (not more than 12 months since the most recent attempt) and “in early remission” (12–24 months since the most recent attempt). The criteria also explicitly define “suicide attempt” as “a self-initiated sequence of behaviors by an individual who, at the time of initiation, expected that the set of actions would lead to his or her own death” [(8), p. 801]. This definition emphasizes the importance of intent when defining suicidal behavior while also recognizing the dilemma that individuals' ratings of suicidal intent do not always match the absolute or understood lethality of their methods of attempted suicide (10). The diagnosis of SBD is also explicitly differentiated from another condition for further study, “Non-suicidal Self-Injury.” These criteria provide a helpful start for the investigation of such a disorder, but criteria could and should be refined with additional research into the construct.

### Guidelines for *SBD* Evaluation

The *DSM* task forces evaluated SBD using the same “Guidelines for Making Changes to *DSM-V*,” which were used to evaluate all *DSM-5* diagnoses (9). Encompassing and elaborating upon the recommendations of Robins and Guze (11) for the establishment of diagnostic validity, these guidelines provide information for how the *DSM-5* work groups should make decisions about diagnosis validity and clinical utility. Throughout this discussion we will use these guidelines to highlight potential support of or concerns with inclusion of SBD in a future *DSM*.

First, the guidelines provide the validator categories by which a disorder's research should be evaluated. Kendler et al. divide this list into three over-arching categories: antecedent validators (i.e., familial aggregation and/or co-aggregation, socio-demographic and cultural factors, environmental risk factors, and prior psychiatric history), concurrent validators (i.e., cognitive, emotional, temperament, and personality correlates; biological markers; and patterns of comorbidity), and predictive validators (i.e., diagnostic stability, course of illness, and response to treatment). They designated several validator sub-categories as high priority: familial aggregation and/or co-aggregation, diagnostic stability, course of illness, and response to treatment. They pronounced that any new diagnosis should have a substantial amount of research supporting the disorder across the validator categories, with research particularly focused in the high priority validator categories and with at least some research of high methodological quality. While limited research has been performed on SBD specifically, research on suicide attempts and suicide in general is extensive and can be applied to our understanding of SBD.

Second, Kendler et al. (9) provided additional concerns about clinical utility for including new diagnoses (as compared to changing previously existing diagnoses). They identified five considerations: a need for the category, relationship to other *DSM* diagnoses, potential harm, available treatments, and meeting criteria for a mental diagnosis. They also stressed the importance of diagnosis reliability. Through these and other considerations, they argued that any addition to the *DSM* requires a comprehensive explication of the advantages and disadvantages of a proposed diagnosis. While researchers had previously argued that the inclusion of a suicide disorder in the *DSM* would be valid and useful [e.g., (12–14)], few articles have examined the validity and utility of SBD criteria since the release of the *DSM-5*. Using the guidelines set by Kendler et al., we ultimately intend to argue that SBD largely fits the criteria for inclusion as a DSM diagnosis, though there are related alternative diagnoses or improvements to the DSM that should be considered beyond SBD due to the potential limitations of SBD as it is currently proposed.

## Diagnostic Validity of SBD

### Antecedent Validator: Familial Aggregation and Co-aggregation

The first-listed antecedent validator for a suicide diagnosis, familial aggregation and co-aggregation, is a high-priority validator category that refers to the extent that genetics influences a disorder as determined by evidence from family, twin, or adoption studies. Literature reviews support the notion that suicide clusters in families and is genetically influenced (15–17). There are a number of studies revealing aggregation of suicide in families (18). Two of which, notably, examined large national death registries in different countries (19, 20), and found family history of suicide to be a significant predictor of suicide. In one of these studies (20), familial suicide rates were twice as high in individuals who died by suicide as compared to individuals who died by other causes. Twin studies of various methodologies have sustained the genetic influence over suicide, finding that monozygotic twins have higher rates of suicide attempt and completed suicide concordance than dizygotic twins (17, 18). Importantly, family and twin studies have found that the familial transmission of suicidal behavior goes above and beyond transmission of risk for psychiatric illness in general (20–23). Ultimately, heritability of suicidal behavior ranges between 38 and 55% (18), and between 17 and 36% when controlling for other psychiatric illness (24). These heritability rates are similar to other already-validated disorders in the DSM-5 [e.g., MDD's heritability rate is reported as ~40% (8)].

Even though there is clear relevance of genetic vulnerabilities for suicide, it can be challenging to disentangle these risks from shared family environment risks. For example, suicide appears to have a contagion effect, such that individuals sometime seem more likely to engage in suicidal behavior after becoming aware of others' suicidal behavior (25). While it might be expected that familial influence over suicide could be related to imitation rather than genetics, research challenges this notion. First, in research examining the role of suicide imitation in families, there is no significant temporal relationship between suicidal behaviors in relatives (18). Second, a number of adoption studies have found a strong role of genetics for suicidal behavior (16, 18), eliminating the possibility of familial imitation. While heritability of suicide is certainly affected by the heritability of psychiatric illness (26) and other heritable traits [e.g., impulsivity (24)], the overall literature suggests a familial aggregation of suicidal behavior distinct from familial imitation and inheritance of psychiatric illness.

### Antecedent Validator: Environmental Risk Factors

The literature also reveals the importance of epigenetics and a variety of environmental factors on risk for suicide, and SBD is also supported by clear environmental precipitants to behavior. Research in this area has been extensive, and there are a variety of both long-term and short-term risk factors. One of the most significant long-term risk factors for suicidal behavior is early life adversity. Suicidal behavior is associated with childhood emotional neglect or physical abuse, parental death or illness, and childhood sexual molestation or rape (24, 27, 28). Furthermore, there appears to be a dose-response effect, with greater amounts of stressful events leading to greater amounts of risk of suicide (29). Another strong environmental risk factor for suicide is access to lethal means. Growing evidence suggests that, in the United States, states with stricter firearm ownership (e.g., background checks or mandatory waiting periods) demonstrate lower suicide rates and trajectories than states with fewer restrictions (30, 31). Other significant, proximal risk factors include social stressors, including but not limited to facing legal difficulties, being fired from a job, ending of intimate relationships, or being exposed to others' suicidal behaviors (25, 27, 28, 32). Relatedly, there are a number of environmental protective factors for suicidal behavior, including social support and a relationship with a therapist (33). While research suggests that environmental risk factors can change across the lifespan [e.g., with bullying being a particular risk factor in children and adolescents (34)] or differ between sub-groups of people [e.g., with discrimination being a particular risk factor in sexual and gender minorities (35)], the environment undoubtedly impacts risk of suicide attempts.

### Antecedent Validator: Socio-Demographic and Cultural Factors

Beyond environmental risk factors, several socio-demographic, and cultural risk factors for suicide have been identified. Most significantly, suicide risk varies by gender, age, ethnicity, and sexual orientation. Men die by suicide much more frequently than women (28, 32, 36), although women seem to engage in more non-fatal suicidal behaviors (37–39). Transgender individuals (regardless of gender identity) seem to be at particularly increased risk of suicidal behavior, with up to 43% of transgender people reporting lifetime suicide attempts (40). Across genders, most suicides occur between the age of 35 and 44, and suicidal behaviors are very rare before puberty (28). More recent data suggest that risk for suicide could be increasing more rapidly in younger adult cohorts (41). Age-related risks of suicide also seem to differ across ethnicity, with African-Americans and Latino-American more likely to die by suicide when they are younger as compared to White Americans (42, 43). African-Americans, as well as Asian Americans and Native Americans, have lower overall rates of suicide as compared to White Americans (32, 36), although some evidence suggests African-Americans might be more likely to die by suicide at their first attempt (44). Notably, the gender-identity-gap lessens in certain ethnicity groups, with some female racial minorities (e.g., Native American female adolescents) being at greater risk than their male counterparts (32). Finally, sexual minorities (e.g. those who identify as lesbian, gay, bisexual, queer, or non-heterosexual in some way) have significantly elevated risk of suicidal behaviors across the lifespan (45, 46).

In addition to suicide risk being different between certain demographic groups, there are unique cultural risk factors in certain groups. These cultural-specific risk factors include acculturation, collectivism vs. individualism, religion/spirituality, different manifestations or interpretations of stress, and underutilization of mental health services (32). Culture also seems to influence what other risk factors predict suicide most strongly, with social stressors predicting suicide more strongly than mental illness in East Asia as compared to Western countries (47). While there is growing evidence that suicide risk differs substantially between cultural groups, more research is needed to elucidate these variations (48).

### Antecedent Validator: Prior Psychiatric History

The fourth and final antecedent validator category is prior psychiatric history. Psychopathology is highly associated with suicide risk (12, 24, 28, 32), and ~80% of American suicide attempters had temporally prior diagnosed psychiatric illnesses (49). Specifically, suicidal behaviors have been associated with depression (49–51), anxiety disorders (12, 24, 49, 52), substance use (49, 50), bipolar disorder (28, 50), eating disorders (53), schizophrenia (54, 55), and personality disorders (56, 57). Childhood impulsivity, state-like agitation and anxiety, and lifetime difficulties with aggression (in the form of conduct or antisocial disorders) are also related to suicidal behaviors (27, 49, 50, 58). The *DSM-5* discusses suicide risk in the context of many psychiatric disorders, and the literature suggests that prior psychiatric history is paramount in determining suicide risk.

### Concurrent Validator: Cognitive, Emotion, Temperament, and Personality Correlates

In an attempt to understand suicidal behavior and increase clinicians' ability to predict it, a vast amount of research has focused on concurrent psychological correlates of suicidal behavior. Hopelessness and pessimism for the future have been extensively associated with suicidal thoughts and behavior even when controlling for depression (27). Rumination, a cognitive process in which people repetitively focus on negative feelings and problems, is linked to suicidal thoughts and attempts (59). People who attempt suicide suffer from certain cognitive limitations, including decreased problem-solving skills (60), decreased verbal fluency (61), and decreased ability to recall autobiographical memories (62). Suicide attempters also show elevated attention to (and interference by) suicide-related stimuli on stroop tasks (63), as well as significant implicit associations between self-concepts and death-related words and imagery on Implicit Association Tests (64).

Beyond cognitive validators, suicide is related to various emotional, temperamental, and personality factors. The use of suppression as an emotion regulation strategy is associated with suicidal behaviors and may mediate the relationship between emotional reactivity and suicidal behavior (65, 66). Impulsivity and aggressiveness seem related to suicide (27, 32, 58). Additionally, perfectionism, neuroticism, introversion, and other personality facets have been connected to suicidal behavior (67–69). More research is needed to further substantiate whether these cognitive and personality factors are predictive of suicidal attempts, but it is clear that there are a number of psychological correlates of suicidal behavior.

### Concurrent Validator: Biological Markers

Research in the area of the second concurrent validator, biological markers, is in its relative infancy but is very promising. While more research is needed to confirm potential biomarkers, evidence suggests that many neurobiological systems are related to suicide, most notably the stress response system and the serotonergic system (24, 28, 70–74). For example, a hyperactive stress response, as revealed *via* a dexamethasone suppression test, has been found to be related to suicide attempts (75–77) and may even be predictive of future suicide attempts (78, 79). Furthermore, suicidal behaviors are associated with low serotonin and serotonin metabolites in spinal fluid and blood (80, 81). Low levels of 5-hydroxyindole acetic acid, the primary metabolite of serotonin, may be another potential predictive biomarker for suicide attempts (75). Finally, there are a number of possible genetic markers of suicidal behavior (16). Primarily, there certainly are biological correlates to suicidal behavior, but more research is needed to understand how exactly these biological systems and biological markers could aid clinicians in the identification and treatment of suicidal patients.

### Concurrent Validator: Patterns of Comorbidity

Most of the relevant research to the final concurrent validator, patterns of comorbidity, overlaps with the antecedent validator of prior psychiatric history. As described above, suicidal behavior can occur in the context of many psychiatric disorders, although certain disorders have particularly strong relationships with suicidal behaviors. MDD and BPD, for example, include suicidality as a part of their diagnosis criteria, partly because these disorders so often occur co-morbidly with suicidal ideation and suicidal behavior. Impulsivity (which is often experienced in BPD or substance use disorders) and agitation (which is often experienced in disorders like Post-Traumatic Stress Disorder) have also been uniquely correlated with suicidal behavior (49, 50). Therefore, there are certain disorders that likely would occur comorbidly with SBD more often than other disorders, although research on SBD would be needed to confirm this assumption.

### Predictive Validator: Diagnostic Stability

The first of the predictive validator categories, a high-priority category, is diagnostic stability. Diagnostic instability may be related to the evolution of an illness, emergence of new information, or measurement unreliability [(82), as cited in (83)]. At face level, it would be expected that SBD would have very high diagnostic stability within a certain time period, given that the diagnosis criteria are written to dichotomously capture the presence of a single behavior in the past 2 years. After that 2-years time period, however, the person abruptly would no longer meet criteria for SBD if they have not had any further suicide attempts. Additionally, consistent identification of the disorder would require reliability of its assessment. Therefore, when considering SBD as a potential diagnosis, its diagnostic stability should be evaluated by the reliability of assessment of suicidal behavior and by suicide behavior's relative persistence over time.

#### Reliability of Diagnosis

Reliability is an issue related to diagnostic stability that likely contributed to SBD's exclusion as a valid disorder in the *DSM*-5. Kendler et al. (9) explicitly recognize reliability as being important when considering new diagnoses and that they “would not expect to support the addition of new diagnostic entities in *DSM-V* [*sic*] without some evidence that they are [at least moderately] reliable” (p. 7).

The field of suicidology is plagued by inconsistent nomenclature, and the validation of structured interviews of suicidal behavior is still developing. Nevertheless, proper assessments of suicidal behavior and suicide risk exist. For example, the Columbia—Suicide Severity Rating Scale (C-SSRS) shows promise as a valid and reliable in-person (84–86) or computer-automated (87) assessment of overall suicide risk by assessing suicidal ideation, planning, intent, and actions (84). Another measure, the Self-Injurious Thoughts and Behaviors Interview (SITBI), has been used fairly extensively as a valid and reliable measure of non-suicidal and suicidal self-injurious features (88). Since the publishing of *DSM-5*, Fischer et al. (89) operationalized the SITBI items into the criteria for SBD. They found that their version of the SITBI had moderate to good test-retest reliability for current SBD (κ = 0.52) as well as perfect interrater reliability for SBD. Therefore, it appears that SBD could have sufficient reliability as a diagnosis.

Past measures, however, largely have been validated to assess and determine both suicidal behaviors and thoughts or overall suicide risk, rather than suicidal behavior exclusively. Even the Fischer and colleague's SITBI assessment of SBD involved simultaneously assessing for NSSI and the proposed NSSI Disorder. This ability to differentially diagnose NSSI and suicidal behaviors might be paramount in ensuring the reliability of a given assessment. When research examines assessment of suicidal behavior specifically, reliability is problematic. People who are asked about suicide attempt history using one-item assessments commonly used in research (e.g., “Have you ever attempted suicide?”) often respond inaccurately (90, 91). In one study, 984 US military service members at risk of suicide were asked about their history of suicide attempts using five previously validated measures (including the C-SSRS), and 35% of participants inconsistently responded across measures (92). This inconsistency is concerning, particularly in the context of SBD's criteria as they are currently written (i.e., with an exclusive focus on suicidal behavior). Given the poor reliability of suicide behavior assessment demonstrated in previous literature, large-scale replication of Fischer et al.' study is warranted in order to solidify the reliability of assessments of SBD specifically.

#### Suicidal Behavior Persistence

While there is no data available yet about how stable a diagnosis of SBD is across time, we can extrapolate the stability of SBD from the data on the persistence of suicidal behavior. Research has demonstrated consistently that the absolute strongest predictor of future suicide attempt is a past suicide attempt (93). Accordingly, studies have found anywhere from 18.9% ((94)) to 88% (95) of people who attempt suicide will attempt again. Rates of re-attempt appear to differ by age, gender, psychiatric diagnosis, and severity of first suicide attempt method (94–97), but more research is needed in this area to confirm patterns. Notably, the 2-years window in SBD's diagnostic criteria is supported by this area of research, with numerous studies suggesting that risk for re-attempt is highest within the 2 years after a suicide attempt (95, 98–100). Some research suggests that risk for re-attempt is highest within the 1st year after an attempt (94, 96, 101), or immediately upon discharge from psychiatric hospitalization (102). One recent study found that 23% of people who presented to an emergency room for a suicide attempt re-presented for a subsequent suicide attempt within 90 days (103). Despite the particularly increased risk immediately following an attempt, increased risk for repeated attempts persists for decades. In one study, about two-thirds of suicide deaths of people who had previously attempted occurred at least 15 years after the first noted suicide attempt (104).

It should be noted that determining the persistence of suicidal behavior is partly hindered by the fact that the majority of people who die by suicide die during their first attempt. In one large study using the National Violent Death Reporting System, 79% of the identified 73,490 people who died by suicide from 2005 to 2013 died on their first suicide attempt (44). Similarly, in a longitudinal study of 813 community youth aged 10 to 24, 29 participants (3.9%) died by suicide and accounted for 90% of the deaths in the sample. Of these, 20 participants (71%) died at their first attempt (105). Of course, many people who attempt suicide do not make additional attempts, and therefore the diagnosis of SBD may not be stable across many years. Research on the diagnostic stability of SBD specifically is needed. A lack of long-term diagnostic stability of SBD might not reflect lack of validity of the diagnosis *per se*, but rather the time-limited and dichotomous nature of its diagnostic criteria requirements. Further, a lack of diagnostic stability could be acceptable given past debate about the value of diagnostic stability as a validity determinant (83). Kendler et al. (9) list diagnostic stability as high-priority, however. Therefore, based on the current literature, and given the reliability concerns for suicidal behavior assessment, diagnostic stability is the validator for which SBD's evidence is currently most weak.

### Predictive Validator: Course of Illness

The predictive validator of “course of illness” arguably has limited applicability to SBD in its current proposed form, given the inherent time constraints of the diagnostic criteria. Again, despite the lack of research on SBD's course of illness, applicable information can be gleaned from general research on suicide.

As already mentioned, suicide attempts predict later suicide attempts, and this risk varies predictably based on frequency and time. The number of times a person has attempted suicide is positively correlated with future suicide attempts, with repeated attempters having up to double the risk of future attempts as compared to people who have attempted only once (55, 106). Conversely, as highlighted previously, amount of time since attempt negatively correlates with risk of future suicide attempt. An individual's risk of re-attempting suicide is highest immediately following an attempt or following discharge from an attempt-related hospitalization (55, 107, 108), and particularly increased risk continues up to 2 years (95, 98). In one study, while risk was highest immediately after an attempt, the vast majority (82%) of suicide attempts who went on to die by suicide did so within a year of their first suicide attempt (93). The “current” specifier of SBD is grounded in and validated by this evidence.

Despite the particularly heightened risk immediately after an attempt, suicide attempt risk continues for much longer. Numerous longitudinal studies reveal that suicide attempts accumulate over time, and that risk for repeated suicide attempt continues for many years and even decades after index attempts (55, 99, 109–111). Therefore, the risk for suicide attempt continues regardless of time after one suicide attempt. The course of illness of SBD would likely mirror this pattern.

The *DSM-5*'s current description for SBD's course of illness states, “there is significant variability in terms of frequency, method, and lethality of attempts” (p. 802). While this claim is true, course variability is seen in other *DSM* disorders (e.g., depression, psychosis) and would not be unique to SBD. Further, while attempted suicide can look incredibly different between different people, there is some data to suggest that suicidal individuals might use methods of similar type and lethality across multiple attempts (99, 112), implying at least some intra-person consistency of course of illness of suicide attempts. More research is needed to fully describe the course of illness of SBD specifically, and perhaps specifiers related to method, lethality, or number of previous attempts should be considered and examined.

### Predictive Validator: Response to Treatment

The final predictive and final high-priority validator is “response to treatment.” There are several reviews of suicide literature that suggest suicidal behaviors can be reduced with various treatments and prevention measures (24, 28). Both medications [e.g., clozapine and lithium (113, 114)] and talk therapies [e.g., Dialectical Behavior Therapy (115, 116) and Cognitive Behavioral Therapy (117)] have been found to decrease suicidal behaviors in certain populations. In deeply depressed, acutely suicidal individuals, electroconvulsive therapy reduces subsequent suicidal behaviors (118). Noteworthy here is that many treatments targeting depression specifically do not impact suicidal thoughts and behaviors (119), suggesting some specificity in response to treatment for SBD. Finally, research suggests that suicide attempters are less likely to later die by suicide if upon discharge they are scheduled to have follow-up attention or treatment after hospitalization (93, 103, 120), suggesting that future suicide attempts could be prevented if treatment were scheduled or given to individuals immediately after being diagnosed with SBD.

### Review of Validators

Kendler et al. (9) suggested that any new *DSM* diagnosis should have substantial and consistent support across a variety of validators, and most importantly should have evidence in areas concerning familial aggregation, diagnostic stability, course of illness, and response to treatment. Previous research reveals that suicide attempts (and therefore SBD diagnoses) most definitely aggregate in families (as determined *via* family, twin, and adoption studies), have a specific course of illness (with risk of future suicide attempt being most intense immediately after one attempt but persisting over the lifespan), and have responsiveness to treatment (with several medical and psychosocial treatment options). The literature also supports other validators, including cultural factors, environmental risk factors, past psychiatric history and comorbidity patterns, concurrent correlates, and biological markers. While research demonstrates some possible diagnostic stability (in the form of continued risk for suicide after initial suicide attempt), there are significant, possible concerns related to reliability of SBD's assessment. Nonetheless, considering there is preliminary research on SBD assessment reliability that has surfaced since the *DSM-5*'s publishing (89), we argue that SBD demonstrates substantial diagnostic validity based on the current literature on suicide attempts, although further research is needed to solidify its validity. The greatest issues with SBD, reliability-related and otherwise, concern its clinical utility.

## Clinical Utility of SBD Considerations

Kendler et al. (9) stipulated that, beyond demonstrating empirical validity, any new *DSM* diagnosis should have clinical utility illustrated through comprehensive debate of the diagnosis' benefits and potential costs in five areas. Some have argued elsewhere that SBD would provide significant clinical utility [e.g., (121)], while others have highlighted several significant limitations or concerns [e.g., (122)]. We present below further evidence and arguments related to the five clinical considerations: need for the category, relationship to other diagnoses, potential harm, available treatments, and meeting criteria for a mental diagnosis.

### Consideration 1: Need for the Category

The first consideration to contemplate when debating the inclusion of a new diagnosis into the *DSM* is the need for the category, or the extent to which a new diagnosis would help clinicians be more aware of and treat a distinct group of people who may not be served under current diagnoses. Arguably, a new diagnosis is not needed if it does not improve patient care. We maintain that SBD offers considerable benefit. A large proportion (24–66%) of individuals who die by suicide are in contact with a mental health provider within the year before their death (107, 123, 124), and approximately half of individuals who die by suicide have previously self-harmed [(125), as cited by (126)]. While it is still unclear if and how these deaths by suicide could be prevented by contact with mental healthcare, research is unequivocal about the fact that healthcare providers are habitually under-trained in suicide risk assessment (127, 128).

While there has been an increase in required suicide risk assessment in hospital systems and healthcare clinics in the past decade, lack of confidence in suicide risk assessment training persists in healthcare workers, including clinical psychology graduate students (129), nurses (130), and medical residents (131). This under-training likely negatively influences clinical care. Even in systems that explicitly emphasize the necessity of suicide risk assessment, clinicians ask about self-harm inconsistently across patients (132). When clinicians do assess self-harm, they may ask questions in ways that decrease the likelihood of honest answers [e.g., with negativity bias; (133)]. Similarly, clinicians who report receiving comprehensive training in suicide risk assessment may still routinely miss key questions in risk assessment [i.e., not asking about multiple previous attempts, or not asking about lethal means used in previous attempts; (134)]. These problems could be addressed with improved training, and others have argued how clinician training would be greatly improved by the development of guidelines on how to deliver and assess trainings on suicide risk assessment (135). Clearer guidelines, in turn, would be easier to implement with an agreed-upon definition and assessment of suicidal behavior, such as one that could be provided by SBD.

SBD's presence also could inherently increase the amount of time spent assessing suicide in clinical intakes. Currently, as mentioned previously, suicide is included in the *DSM* only as a symptom of MDD and BPD. In many current semi-structured assessments, if a client denies experiencing major difficulties with depressed mood and anhedonia, the clinician likely would not ask the remaining MDD questions (including questions about suicidal ideation); and if the client does not report intense emotion dysregulation or interpersonal difficulties, the clinician may not assess BPD (including questions about self-harm). In these cases, it is possible that the assessor would ask no questions about suicidality. Even if MDD is present and suicidal ideation is assessed, clinicians may not ask about suicidal behaviors. Therefore, in some cases, suicide risk determination may be incorrect due to lack of assessment of suicidal behavior, and certain individuals who are at risk for attempted suicide may be entirely missed.

Of course, based on previous training or specific clinic guidelines, some clinicians may include suicide assessment outside of the *DSM* diagnoses of MDD and BPD. Without accepted guidelines or standardized measurements, however, assessments differ greatly between clinicians. Many current measures of suicidality include single items about suicide, or use terminology without defining it, causing the very real possibility of client misinterpretation of what the clinician is asking (90, 136). There is also the possibility that, without standardized measures, clinicians ask about suicidal behaviors in ways that are pejorative (137) or in ways that discourage certain people (e.g., ethnic minorities) from accurately reporting (48). Furthermore, clinicians often disagree about what types of behavior to include in “suicide attempt” vs. “non-suicidal self-injury,” “aborted attempt,” and “interrupted attempt.” These separate concepts have differential impact on suicidal risk (50), and confusion about their distinctions can have negative impacts on clinical care (90, 138). SBD's inclusion would help to create standardized nomenclature, which would improve both assessment of suicide risk and communication of risk between treatment providers (120, 137). In order to fully address this clinical need, however, the *DSM* might also need to provide a suggested, validated measure of SBD, rather than just the diagnostic criteria. We discuss this idea, including validated suicide risk assessment in the *DSM*, more fully below.

Beyond assessment, SBD as a specific diagnosis could improve outcomes, given the particularly increased risk of re-attempt in the immediate after-math of an attempt. For example, hospital systems could use the diagnosis of SBD in electronic medical records to flag significantly at-risk patients to then receive heightened follow-up attention or specific treatment referrals. Initial research demonstrates actions like these might be useful in preventing subsequent suicide attempts (103, 139–141). Finally, SBD's creation of consistent suicide terminology would positively impact clinical work *via* research. If clinical assessments of attempted suicide were more precise and universal, studies of attempted suicide in turn could become more precise and larger-scale, which in turn would allow more accurate findings about risk factors for attempted suicide and identify more features for clinical targets (90, 142).

Overall, the *DSM* can have immense impacts on research, clinical care, and public health (143). The inclusion of SBD would implicitly communicate the importance of suicide assessment. It would provide large logistical advantages to research by creating an accepted nomenclature and by increasing the amount that suicide attempts are captured in health records. It would benefit clinical care by increasing clinician awareness, improving inter-clinician communication about suicide behavior history, and increasing the likelihood that clients with past (and potential future) suicide attempts would be recognized and treated appropriately.

### Consideration 2: Relationship With Other *DSM* Diagnoses

In the second consideration, Kendler et al. (9) emphasize the importance that any new diagnosis should be sufficiently distinct from other *DSM* diagnoses. While no research to our knowledge has examined SBD's comorbidity with other disorders, some arguments about SBD's separateness as a diagnosis can still be made. One study examining the diagnostic profiles of suicide attempters upon hospital discharge, for example, found that suicidal behavior most frequently occurred within alcohol use disorder (34% of the sample), depression (16%), and schizophrenia (10%), with depression being the diagnosis most common in those who re-attempted within 30 days of discharge [32%; (144)]. In accordance with this finding, suicidal behavior has been connected most to MDD and BPD in their etiology, risk factors, and patterns of comorbidity. While research shows strong relationships between suicide and these disorders, it also suggests important distinctness. Evidence suggests that while depression predicts suicidal ideation, it does not predict suicidal behavior (49), and the majority of depressed people do not engage in suicidal behaviors (145, 146). Moreover, treatments targeting depression specifically do not necessarily decrease suicidal behaviors (119), and depression and suicide attempts may even have distinct neurobiological influences (147). Similarly, not all individuals with BPD report suicidal behaviors (148, 149), and many individuals who attempt suicide do not suffer from either MDD or BPD (121, 150). While MDD and BPD do correlate with suicide attempts, this relationship can disappear when controlling for previous suicide attempts (151).

Beyond MDD and BPD, suicide attempts also occur in the context of schizophrenia, substance use disorders, anxiety disorders, eating disorders, and other personality disorders (144). SBD's likely common comorbidity with other disorders would be no different than the high rates of comorbidity elsewhere in the DSM. For example, BPD heavily co-occurs with mood disorders [76%; (152)], substance use disorders (73%), and other personality disorders [74%; (153)]. Similarly, anxiety disorders co-occur with depressive disorders up to 80% in certain samples (154). These types of patterns expand across diagnoses, with 79% of psychiatric disorders occurring with some lifetime psychiatric comorbidity (155), and more than half of people diagnosed with psychiatric disorders in the past 12 months having more than one disorder (156). Of course, more research on SBD specifically, rather than on suicidal behavior, is needed to confirm the assumption that SBD's rates of comorbidity would mirror those of other diagnoses. Regardless, it's important to note that SBD would provide unique diagnostic information, given that SBD's symptomatology overlaps exclusively with BPD, the only diagnosis to include criteria about suicidal behavior specifically.

Not all individuals struggling with psychopathology engage in suicidal behavior, and, more importantly, not every person who attempts suicide struggles with psychopathology (121) or has previously diagnosed psychiatric disorders (44, 49). In one decades-long study of medical records at a large Minnesota hospital, 41% of community youth who died by suicide had no mental health diagnosis prior to their first attempt (105). In another study of 273 psychiatric patients hospitalized for suicide attempt in France, 4% of participants did not meet diagnostic criteria for any disorder according to MINI interview at time of hospitalization (98). These findings mirror decades of psychological autopsy studies that have found that, while the large majority of people who die by suicide have a mental disorder of some sort, there remain a proportion of suicide decedents who do not (157). Of course, much of this research is hindered by retrospective, self-report, or posthumous data. Some have argued that these findings might be due to methodological flaws or clinical errors, and that suicide only occurs within mental health disorders and issues (158, 159). Others, however, continue to assert that suicide happens outside of mental illness, particularly in response to intense social stressors or particularly in non-Western countries (47, 160–162). In accordance with those arguments and existing evidence, SBD would best be considered a distinct disorder, in spite of its potential comorbidity with other DSM disorders.

### Consideration 3: Potential Harm

Perhaps the most controversial consideration for SBD is the consideration of potential harm to affected individuals or to broader society that the inclusion of a new disorder could create. Others have argued that the inclusion of SBD could potentially over-medicalize a symptom (163). There's a general recognition that psychiatry is increasingly turning public health problems (e.g., suicide, internet gambling addictions, substance use) into disorders in a way that may over-simplify very complex human behaviors. Medicalizing a behavior like suicide could arguably increase the likelihood that a behavior like homicide would be medicalized, which certainly could have negative consequences in the legal system and society as a whole. While it is of course important to consider the impact SBD's inclusion could have on the inclusion of other “problem behaviors” as disorders, these potential disorders (e.g., a disorder for homicide) would and should be evaluated separately from SBD, and therefore should not be large considerations in SBD's evaluation. Furthermore, SBD would not be the first disorder to “medicalize” behaviors, and medicalization does not seem to be a particular concern to the *DSM*. Similarly, SBD would not be the first disorder in the *DSM* based on the presence of behavior, rather than the “syndrome” model and collection of co-occurring symptoms typical of most disorders. Encopresis has been included in multiple *DSM* versions, for example, and the *DSM-5* includes disorders for binge-eating and fire-setting (8). Evidence that supports the notion that over-medicalization and over-diagnosis of behaviors is harmful remains limited (164).

Of course, there is the possibility patients could be over-pathologized or stigmatized for “an expression of distress” (p. 857) in the form of self-injury, if SBD (and NSSI Disorder) are included in a future *DSM* (163). This concern is very important. Receiving a diagnosis of SBD could very well limit a patient's options in providers, as many healthcare clinicians are uncomfortable working with suicidal clients. Yet, this limitation of clinicians might also ensure clients are only referred to programs or clinicians most competent to help them, as often occurs with patients diagnosed with BPD and substance abuse disorders (which are also stigmatized). Receiving a diagnosis of SBD might also stigmatize a person who is otherwise “mentally healthy.” As previously discussed, some individuals who attempt suicide might not meet criteria for any other mental health diagnosis. Indeed, some people who attempt suicide might do so within the context of psychic distress caused by extreme social stressors (e.g., job loss, chronic bullying, or racial victimization). Yet, while a traumatized person's distress and desire to attempt suicide could be understandable, turning to suicidal behavior in distress should be clinically considered separately and often should be considered to be problematic (as we will argue further below). Notably, SBD does not pathologize thinking about suicide. A suicide-attempt-related diagnosis like SBD might increase the ability for healthcare systems to provide important treatment and support to a marginalized person in intense distress after they have attempted, by focusing the diagnosis on the problem behavior of suicide without further medicalizing or stigmatizing the person's understandable emotional reaction to extreme life circumstances. Finally, as previously highlighted, SBD inclusion might increase population levels of clinician training in (and therefore comfort with) suicide behavior assessment and treatment, which would benefit all people presenting to healthcare systems with suicidal behavior. Generally, we believe the inclusion of SBD as a diagnosis would improve awareness and management of suicide risk, as argued above, in a way that out-weighs most potentials for harm that have been most commonly identified and argued in the literature thus far.

In our view, the largest problem of SBD's potential harm relates to its singular focus on suicidal behavior, the reliability of suicide behavior assessment, and the complexity of suicide risk determination. Assessments that exclusively assess suicide behavior, or assess suicidal symptoms using one-item measures, are more likely to be answered inaccurately or inconsistently (90). While previous suicide attempts are the strongest predictor of a future suicide attempt (4), the most accurate suicide risk assessment involves assessment of a variety of components beyond past behavior. SBD's inclusion might increase clinician assessment of suicide behavior in their patients, but SBD's focus on history of suicidal behavior could lead to clinician over-reliance on past suicidal behavior information in their risk assessments. It could also lead to under-identifying people who are at risk for suicide despite having no history of attempts. To be most clinically useful with less chance of harm, therefore, SBD could explicitly include other suicide-related criteria, such as history of suicide preparation behaviors, history of aborted suicidal attempts, and/or current or recent suicidal intent or ideation. These types of changes would make SBD represent more of a “syndrome” of suicidal behaviors, rather than relying on a dichotomous variable focused on one specific type of behavior. If SBD would be most clinically useful with diagnostic changes, and SBD without these changes could cause harm, however, then the proposed diagnosis of SBD should arguably not be included as it is currently written in *DSM-5*.

### Consideration 4: Available Treatments

The fourth consideration suggested by Kendler et al. (9) is “available treatments.” It could be argued that the inclusion of a new diagnosis would be harmful or at least useless if there were no treatments that could reliably and effectively treat the new disorder. We have already described above in the “response to treatment” validator section that there are a number of treatments and prevention methods that seem to impact and decrease suicide attempts and self-injury in general (24, 28). Therefore, SBD should be evaluated in a positive light when scrutinizing this fourth consideration for clinical utility.

### Consideration 5: Meets Criteria for a Mental Diagnosis

It is important that any new diagnosis meets the general criteria for a mental diagnosis and does not pathologize a normal variation of normal behavior. While Kendler et al. (9) recognize that there is no official definition for mental diagnosis, they reference the definition provided by Stein et al. (165) as a useful one to consider when evaluating potential diagnoses. First, a mental disorder must be “a behavioral or psychological syndrome or pattern that occurs in an individual” that causes “clinically significant distress (e.g., a painful symptom) or disability (i.e., impairment in one or more important areas of functioning)” [(9), p. 6]. Even though suicide does not always co-occur with diagnosed psychopathology, as noted above, many have argued that suicide is always associated with distress and mental health difficulties that could be considered “sub-threshold” for mental health disorders and therefore noteworthy [e.g., (159)]. Suicide attempts often immediately follow (and perhaps are “triggered by”) life stressors, such as interpersonal conflict, legal problems, debilitating physical illness, or loss of employment (44, 161). Importantly, most people face these types of stressors without engaging in self-harm, even though many people also experience thoughts about suicide in the context of intense emotions; there is an additional level of psychic pain or other symptoms needed for stressful events to lead to suicide. The literature sustains that it is very rare that a person attempts suicide outside of experiencing some “clinically significant distress,” even if that distress is understandable given an individual's current circumstances. Furthermore, suicidal behavior could be argued to inherently be a “disability” as defined above, given suicide's direct negative influence on a person's ability to function by leading to death or injury. Therefore, we argue that SBD meets this feature of mental diagnosis.

Second, Stein and colleagues state that a disorder “must not be merely an expectable and culturally sanctioned response to a particular event” (p. 6). While self-injury and purposeful death or “rational suicide” have accepted places in certain cultures, in certain forms, at certain times (166), suicide is condemned in most cultures. One current area of conflict related to this issue is physician-assisted death or suicide within the context of terminal illness or certain lifelong disability (e.g. as with dementia). This area of debate has grown over the past decade as more US states and countries across the world begin to adopt physician-assisted death laws. The various arguments for and against physician-assisted death, particularly for psychiatric disorders, have been provided elsewhere [e.g., (167, 168)] and are beyond the scope of this review. Based on this literature, however, medical illness “exemptions” from meeting SBD diagnosis should be considered in any included version of SBD in future *DSM* revisions.

Third, the disorder should “[reflect] an underlying psychobiological disturbance” (p. 6). As reviewed above, suicide attempts (and therefore SBD) are associated with a variety of psychological problems and biological dysfunctions, and represent a particularly elevated, clinically notable, and arguably problematic level of psychic distress or mental health disturbance. Fourth, the disorder must “not solely [be] a result of social deviance or conflicts with society” (p. 6). While some individuals might attempt suicide in an effort to communicate disagreement or distress with society, this motivation is only one of many that may inspire individuals to hurt themselves. Fifth, the disorder should have “diagnostic validity using one or more sets of diagnostic validators” and should have “clinical utility (e.g., contributes to better conceptualization of diagnoses or to better assessment and treatment)” (p. 6). It has been argued extensively here that SBD mostly meets these features for mental disorder.

### Review of Clinical Utility Considerations

The inclusion of SBD overall would improve research and clinical care by creating a universal terminology for attempted suicide, and improve treatment for suicidal patients by increasing the likelihood that they are appropriately identified and served in healthcare settings. Yet, SBD's exclusive focus on suicidal behavior could lead to a reliability problem, with an over-reliance on behavior in suicide risk assessment, and to under-identifying at-risk patients. Based on its overall clinical utility and its support in all of Kendler et al.' (9) validators, SBD could be a valid and useful clinical diagnosis to consider in the next *DSM*, pending further validation of its specific diagnostic criteria and its potential assessment measures. It would be most valid and useful, however, if the proposed disorder were edited to include other suicidal behaviors or related factors.

## General Recommendations and Possible Alternatives for Suicide Assessment in *DSM*

As one of the primary diagnostic systems used by clinicians in the field of mental health, we contend that the current *DSM* does a disservice to the field by not providing proper tools for suicide risk assessment. Regardless of whether or not SBD in its current form is included in a future *DSM*, the *DSM-5* could be altered in a number of ways that would address the above-discussed issues related to the assessment, treatment, and prevention of suicide.

### Inclusion of Other Suicide-Related Disorders

While only SBD was included as a proposed disorder in the *DSM-5*, several other suicide-related disorders have been proposed in the literature. Obegi (122) has argued for SBD to be totally reformulated. They suggested three criteria to be considered: (1) presence of suicidal ideation/intent in the past 2 weeks (which could be demonstrated by suicidal behavior, among other symptoms), (2) presence of other suicide-related symptoms (i.e., psychological distress, hopelessness, over-arousal, rigid beliefs about suicide, and readiness to die by suicide) in the past 2 weeks, and (3) exclusion of suicidal thoughts and behaviors sanctioned by society/culture. They also proposed possible subtypes and specifiers that are based in literature on suicide risk research, including specifiers for multiple past suicide attempts or a past-month attempt. This alternative SBD proposal addresses many of the limitations addressed in this paper, while also aligning more with the field's move to prevention-focused lens [i.e., the “Zero Suicide” Model; (6)]. SBD, as it currently is proposed in the DSM, captures only those people who have already attempted suicide, not aiding in the prevention of the many deaths of people who die during their first suicide attempt.

Also in line with the field's move to suicide prevention, two other “presuicidal” disorders have been proposed: Acute Suicidal Affective Disturbance (ASAD) and Suicide Crisis Syndrome (SCS). While they include different symptoms, these two disorders both emphasize diagnostic criteria that might help clinicians identify patients who are most imminently at risk for suicide at time of clinical contact. ASAD criteria include four primary features: a drastic, acute increase in suicidal intent, marked social alienation or self-alienation, hopelessness, and over-arousal (i.e., insomnia, irritability, or agitation). Initial research demonstrates ASAD's validity, reliability, and utility (150, 169). SCS includes five primary components: entrapment, affective disturbance, loss of cognitive control, hyperarousal, and social withdrawal. SCS also has promising initial research supporting it (170, 171). Beyond their ability to catch at-risk patients without suicide histories, these disorders would also provide assessments of suicide risk that could change in real-time with the quick changes in mental state that often accompany suicidal behavior. Inclusion of ASAD or SCS into the *DSM*, pending further research, would provide many and more of the clinical benefits of SBD without some of the above-mentioned limitations.

### Creation of an Additional “Axis” or Suicide Risk Assessment Protocol

Before the release of *DSM-5* and the elimination of the five axes, some researchers argued for inclusion of a “sixth axis” specific to suicide risk (12–14). Although an additional axis no longer is appropriate with the removal of the prior DSM-IV axis system, a final way to improve the *DSM* and its coverage of suicide would be to include a standardized suicide risk level assessment into its pages. This inclusion could fit into the increasingly common suggestion that the *DSM* move into more transdiagnostic dimensional measures of syndromes (172) by providing a way for clinicians to rate their clients on a dimensional scale of suicide risk. Similar to the inclusion of SBD, the *DSM*'s inclusion of a general dimensional measure of suicide risk would increase recognition of currently under-served populations by making suicide assessment more customary for all clients, not just those with MDD and BPD. This measurement could be created in a hierarchical way, such that clinicians could determine overall suicide risk level by evaluating their clients' self-report of self-injurious thoughts and behaviors of different risk levels (173). For example, as past attempted suicides are so predictive of future suicide attempts, a client's self-report past attempted suicide would inherently place that client at higher risk than past or current suicidal ideation would. Models of these types of graded suicide risk assessments are available in the literature [i.e., (174, 175)].

Even if the next *DSM* task force and work groups believe creating an entirely new “axis” or comprehensive scale of suicide risk is unnecessary or problematic, there are ways that the *DSM* can and should be improved. Currently, the *DSM-5* includes one question about suicidal ideation in the “Level 1 Cross-Cutting Symptoms Measure for Adults” included in Section III (8). The question asks clients to rate on a scale of “0 – None – None at all” to “4 – Severe – Nearly every day” “how often have [they] been bothered by” “thoughts of actually hurting [themselves]” in the past 2 weeks [(8), p. 738]. Beyond being a potentially confusing question—for example, what if a person has had thoughts about killing themselves but has not “been bothered” by these thoughts?—this measure item suffers from the same problems with validity and reliability from which other one-item measures of suicide risk suffer (90, 136). Furthermore, several items on the Level 1 measure lead to other specifically recommended questions if a client indicates presence of symptoms. For example, if a patient reports experiencing any level greater than “none” for the question related to “feeling down, depressed, or hopeless,” the *DSM-5* advises that the clinician can use the Level 2 Cross-Cutting Symptom Measure of Depression available online from the APA. The Level 1 suicidal ideation item, however, has no relevant “Level 2” measure to which clinicians can move. Clinicians are consequently left to continue a suicide risk assessment without guidance from the *DSM*, potentially leading to the many problems discussed throughout this review.

Future iterations of the *DSM* should, at a minimum, emphasize the importance of including assessment of suicide risk in every clinical intake and diagnostic evaluation. They also should provide more guidance on other questions that might be relevant for clinicians to consider asking if their client selects a “1” or above on the Level 1 suicidal ideation measure. Previously validated measures, such as the SITBI and the C-SSRS, could be considered. Other empirical guidelines suggest that any suicide risk assessment included in a future *DSM* should consider including: presence of current or recent suicidal ideation, presence of current or recent suicidal intent, presence of current or recent suicidal plans, presence of current or past non-suicidal self-injury, and presence of past attempted suicides; frequency of past non-suicidal self-injury and suicide attempts; and intensity of current or recent suicidal ideation, intent, or planning (14, 33, 138, 142). Additionally, it could aid suicide risk determination to assess a client's confidence in one's ability to make an attempt, current level of hopelessness, current social isolation, and family history of suicide (14). Any of these additional changes to suicide assessment in the next *DSM* would greatly improve clinical care by improving suicide risk assessment and therefore improving treatment of suicidal clients.

## Conclusions

The National Action Alliance for Suicide Prevention (176) claimed that one of the most important steps toward reducing the societal burden of suicide would be to increase the number of people with skills for suicide risk assessment. Certainly, the inclusion of SBD would help reach this goal. While more research is necessary to solidify the evidence for its validators, SBD has a large amount of evidence supporting its diagnostic validity through the current literature on recurrent suicidal behavior. Due to the great importance of suicide as a public health concern and to the relative lack of suicide risk assessment knowledge in our field, SBD also provides clinical utility and benefit. The inclusion of SBD would increase the likelihood that clinicians assess suicide risk beyond the suicidal ideation criterion in MDD and the self-harm criterion in BPD. Furthermore, the inclusion of SBD would provide a universal language that could be used between researchers, mental health clinicians, and general healthcare providers. There are significant limitations to the SBD diagnosis as currently proposed, however. Most notably, it may lack sufficient reliability, and it has the potential to over-pathologize certain individuals who attempt suicide within extremely stressful situations (e.g., terminal illness), and therefore presents some potential for harm. SBD also offers no ability to capture people at risk for attempting suicide for the first time, a recent focus in the field of suicidology. Adding other proposed suicide-related disorders (i.e., ASAD and SCS) or other forms of suicide risk assessment to the *DSM* would help to meet the public health need, while addressing the limitations of SBD. Overall, more research is needed to confirm the validity, reliability, clinical utility, and ethical soundness of SBD or any of the alternative additions introduced in this manuscript. Any suicide-related addition to the *DSM*, however, would improve the field by aiding clinicians in making the best decisions for their clients and ensuring clients at risk for suicide receive appropriate treatment.

## Author Contributions

KF conceptualized, investigated, and wrote the preliminary draft of the review. ES supervised material review conceptualization, manuscript revision, and editing. All authors contributed to the article and approved the submitted version.

## Conflict of Interest

The authors declare that the research was conducted in the absence of any commercial or financial relationships that could be construed as a potential conflict of interest.
